# Supplementary material for: Lessons from a catalogue of 6674 brain recordings
Source: eLife. 2022 Jun 15;11:e66388. doi: 10.7554/eLife.66388 (PMC9200404; doi:10.7554/eLife.66388)
Supplement: Transparent reporting form [file elife-66388-transrepform1.docx]

***eLife’s* transparent reporting form**

We encourage authors to provide detailed information *within their submission* to facilitate the interpretation and replication of experiments. Authors can upload supporting documentation to indicate the use of appropriate reporting guidelines for health-related research (see [EQUATOR Network](http://www.equator-network.org/%20)), life science research (see the [BioSharing Information Resource](https://biosharing.org/)), or the [ARRIVE guidelines](http://www.plosbiology.org/article/info:doi/10.1371/journal.pbio.1000412) for reporting work involving animal research. Where applicable, authors should refer to any relevant reporting standards documents in this form.

If you have any questions, please consult our Journal Policies and/or contact us: [editorial@elifesciences.org](mailto:editorial@elifesciences.org).

**Sample-size estimation**

- You should state whether an appropriate sample size was computed when the study was being designed
- You should state the statistical method of sample size computation and any required assumptions
- If no explicit power analysis was used, you should describe how you decided what sample (replicate) size (number) to use

Please outline where this information can be found within the submission (e.g., sections or figure legends), or explain why this information doesn’t apply to your submission:

This feature article manuscript does not report a new experiment. It reports a re-analysis and evaluation of all existing data from our lab. There is a whole section dedicated to sample size adequacy in our field (see section called ‘Horse two: Low statistical power’).

**Replicates**

- You should report how often each experiment was performed
- You should include a definition of biological versus technical replication
- The data obtained should be provided and sufficient information should be provided to indicate the number of independent biological and/or technical replicates
- If you encountered any outliers, you should describe how these were handled
- Criteria for exclusion/inclusion of data should be clearly stated
- High-throughput sequence data should be uploaded before submission, with a private link for reviewers provided (these are available from both GEO and ArrayExpress)

Please outline where this information can be found within the submission (e.g., sections or figure legends), or explain why this information doesn’t apply to your submission:

Again, we note that this manuscript does not report a new experiment. The whole paper is about reproducibility of electrophysiological effects.

**Statistical reporting**

- Statistical analysis methods should be described and justified
- Raw data should be presented in figures whenever informative to do so (typically when N per group is less than 10)
- For each experiment, you should identify the statistical tests used, exact values of N, definitions of center, methods of multiple test correction, and dispersion and precision measures (e.g., mean, median, SD, SEM, confidence intervals; and, for the major substantive results, a measure of effect size (e.g., Pearson's r, Cohen's d)
- Report exact p-values wherever possible alongside the summary statistics and 95% confidence intervals. These should be reported for all key questions and not only when the p-value is less than 0.05.

Please outline where this information can be found within the submission (e.g., sections or figure legends), or explain why this information doesn’t apply to your submission:

This would be more applicable in a research report than in our feature article. This article is a meta scientific study on research we have conducted over the last 10 years. However, it does report a lot of statistical analysis.

Figure 4 includes 95% confidence intervals around all our “SPNs”. Figure 4B shows that published SPNs were significantly larger than unpublished ones. 95% confidence intervals for this effect are described in the text, next to the independent samples t tests. Figure 5 shows effect size (Cohen’s dz) for all 249 SPNs. Figure 5 also shows observed power and effect size (partial eta squared) for all 40 ANOVAs. Figure 6 shows correlations between SPNs computed in different ways, with effect size R squared.

(For large datasets, or papers with a very large number of statistical tests, you may upload a single table file with tests, Ns, etc., with reference to sections in the manuscript.)

**Group allocation**

- Indicate how samples were allocated into experimental groups (in the case of clinical studies, please specify allocation to treatment method); if randomization was used, please also state if restricted randomization was applied
- Indicate if masking was used during group allocation, data collection and/or data analysis

Please outline where this information can be found within the submission (e.g., sections or figure legends), or explain why this information doesn’t apply to your submission:

This is not applicable to our paper.

**Additional data files (“source data”)**

- We encourage you to upload relevant additional data files, such as numerical data that are represented as a graph in a figure, or as a summary table
- Where provided, these should be in the most useful format, and they can be uploaded as “Source data” files linked to a main figure or table
- Include model definition files including the full list of parameters used
- Include code used for data analysis (e.g., R, MatLab)
- Avoid stating that data files are “available upon request”

Please indicate the figures or tables for which source data files have been provided:

All data used to support this meta-analysis, along Matlab and R scripts and user-friendly guidelines, are available in the complete SPN catalogue on open science framework (https://osf.io/2sncj/). All Figures are compiled from this public resource.

To make it possible for anybody to analyze our data, we developed an app that allows users to: i) view the data and summary statistics as they were originally published; ii) select data subsets, electrode clusters, and time windows; iii) visualize the patterns; iv) export data for further statistical analysis. This repository and app will also be able to accommodate data from future projects. The app is available to download for Windows users at https://github.com/JohnTyCa/The-SPN-Catalogue.
